# Detection of new genetic variants of *Betacoronaviruses* in Endemic Frugivorous Bats of Madagascar

**DOI:** 10.1186/s12985-015-0271-y

**Published:** 2015-03-12

**Authors:** Norosoa H Razanajatovo, Lalaina A Nomenjanahary, David A Wilkinson, Julie H Razafimanahaka, Steven M Goodman, Richard K Jenkins, Julia PG Jones, Jean-Michel Heraud

**Affiliations:** Virology Unit, Institut Pasteur of Madagascar, Ambatofotsikely, BP 1274 Antananarivo Madagascar, Dummy_Only; Centre de Recherche et de Veille sur les Maladies Emergentes dans l’Ocean Indien (CRVOI), Plateforme de Recherche CYROI, 2 rue Maxime Riviere, 97490 Sainte Clotilde La Reunion, France; Madagasikara Voakajy, BP 5181 Antananarivo, Madagascar; Department of Animal Biology, Faculty of Sciences, University of Antananarivo, BP 906 Antananarivo, Madagascar; Association Vahatra, BP 3972 Antananarivo, Madagascar; School of Environment, Natural Resources and Geography, Bangor University, Bangor, Gwynedd United Kingdom

**Keywords:** Coronavirus, Chiroptera, Pteropodidae, Madagascar

## Abstract

**Background:**

Bats are amongst the natural reservoirs of many coronaviruses (CoVs) of which some can lead to severe infection in human. African bats are known to harbor a range of pathogens (e.g., Ebola and Marburg viruses) that can infect humans and cause disease outbreaks. A recent study in South Africa isolated a genetic variant closely related to MERS-CoV from an insectivorous bat. Though Madagascar is home to 44 bat species (41 insectivorous and 3 frugivorous) of which 34 are endemic, no data exists concerning the circulation of CoVs in the island’s chiropteran fauna. Certain Malagasy bats can be frequently found in close contact with humans and frugivorous bats feed in the same trees where people collect and consume fruits and are hunted and consumed as bush meat. The purpose of our study is to detect and identify CoVs from frugivorous bats in Madagascar to evaluate the risk of human infection from infected bats.

**Methods:**

Frugivorous bats belonging to three species were captured in four different regions of Madagascar. We analyzed fecal and throat swabs to detect the presence of virus through amplification of the RNA-dependent RNA polymerase (RdRp) gene, which is highly conserved in all known coronaviruses. Phylogenetic analyses were performed from positive specimens.

**Results:**

From 351 frugivorous bats, we detected 14 coronaviruses from two endemic bats species, of which 13 viruses were identified from *Pteropus rufus* and one from *Eidolon dupreanum*, giving an overall prevalence of 4.5%. Phylogenetic analysis revealed that the Malagasy strains belong to the genus *Betacoronavirus* but form three distinct clusters, which seem to represent previously undescribed genetic lineages.

**Conclusions:**

Our findings suggest that CoVs circulate in frugivorous bats of Madagascar, demonstrating the needs to evaluate spillover risk to human populations especially for individuals that hunt and consume infected bats. Possible dispersal mechanisms as to how coronaviruses arrived on Madagascar are discussed.

## Background

Coronaviruses (CoVs) are enveloped viruses with single-stranded positive-sense RNA belonging to the subfamily *Coronavirinae* in the family *Coronaviridae* (order *Nidovirales*). Genomes of CoVs range from 25 to 32 kb and show high genetic diversity [1]. CoVs are classified into four genera: *Alphacoronavirus*, *Betacoronavirus*, *Gammacoronavirus*, and *Deltacoronavirus* [2].

In mammals and birds, CoVs are associated with upper and lower respiratory illnesses or gastroenteritis. In humans, CoVs infections are commonly caused by HCoV-229E and HCoV-OC43 which generally cause mild respiratory illnesses [3]. A new CoV that causes severe acute respiratory syndrome (SARS-CoV) emerged in humans in 2002–2003 and infected more than 8,000 individuals with mortality rates estimated at around 10% [4]. The emergence of SARS-CoV and its mortality rate have raised the risk of a new pandemic that could threaten public health. For this reason, the scientific community invested considerable interest in the identification and characterization of CoVs especially within mammal reservoirs. Subsequently, two novel human CoVs were discovered: HCoV-NL63 in 2004 [5] and HCoV-HKU1 in 2005 [6]. In June 2012, a third novel coronavirus named HCoV-EMC/2012 (renamed MERS-CoV) was isolated from patients presenting with acute respiratory distress and pulmonary inflammation [7,8].

Studies which aimed to identify potential reservoirs of emerging human CoVs have revealed that the *Betacoronavirus* SARS-CoV was closely related to CoVs detected in bats, specifically members of the genus (*Rhinolophus*), which brought the hypothesis of a spillover of this virus to several animal species (including civet cats and raccoons) sold in Chinese markets as bushmeat for human consumption [9-11]*.* Bats have since become a particular focus and a number of *Alphacoronavirus* and *Betacoronaviruses* have been identified in many frugivorous and insectivorous bat species and in many countries worldwide in Asia, the Americas and Europe (see review from Drexler et al. 2014) [12]. Genomic characterization of the recently discovered MERS-CoV showed that this virus belongs to the genus *Betacoronavirus* and seems to be closely related to bat coronaviruses HKU4 and HKU5 isolated from bats (*Tylonycteris* and *Pipistrellus*) [13].

African bats are known to harbor a range of pathogens (e.g., Ebola and Marburg viruses) that can infect humans and cause disease outbreaks [14-16]. Some authors have reported the detection of bat CoVs from mainland Africa [17-21]. A recent study in South Africa detected a genetic variant closely related to MERS-CoV from *Neoromicia zuluensis*, an insectivorous bat [22]. The authors hypothesize that MERS-CoV may have a common ancestors with CoVs borne by bats from Africa. Though Madagascar is home to 44 bat species (41 insectivorous and 3 frugivorous) of which 34 are endemic [23-25], no data exists concerning the circulation of CoVs in Malagasy bats. Certain bat species on the island can be frequently found in close contact with humans, particularly members of the family Molossidae; these synanthropic species roost in human-occupied buildings, like houses, schools or hospitals [26], whilst frugivorous bats feed in the same fruit trees where people collect and consume fruits. Moreover, hunting and consumption of bat bush meat, especially the larger frugivorous species of the family Pteropodidae, is widespread on the island, bringing hunters, purveyors and consumers into contact with bats [27]. In this study, we report the detection of CoVs amongst frugivorous bats in Madagascar. Our results demonstrated for the first time that CoVs belonging to the genus *Betacoronavirus* are circulating amongst two endemic frugivorous bats species in Madagascar.

## Results

### Virus detection

**Figure 1:**
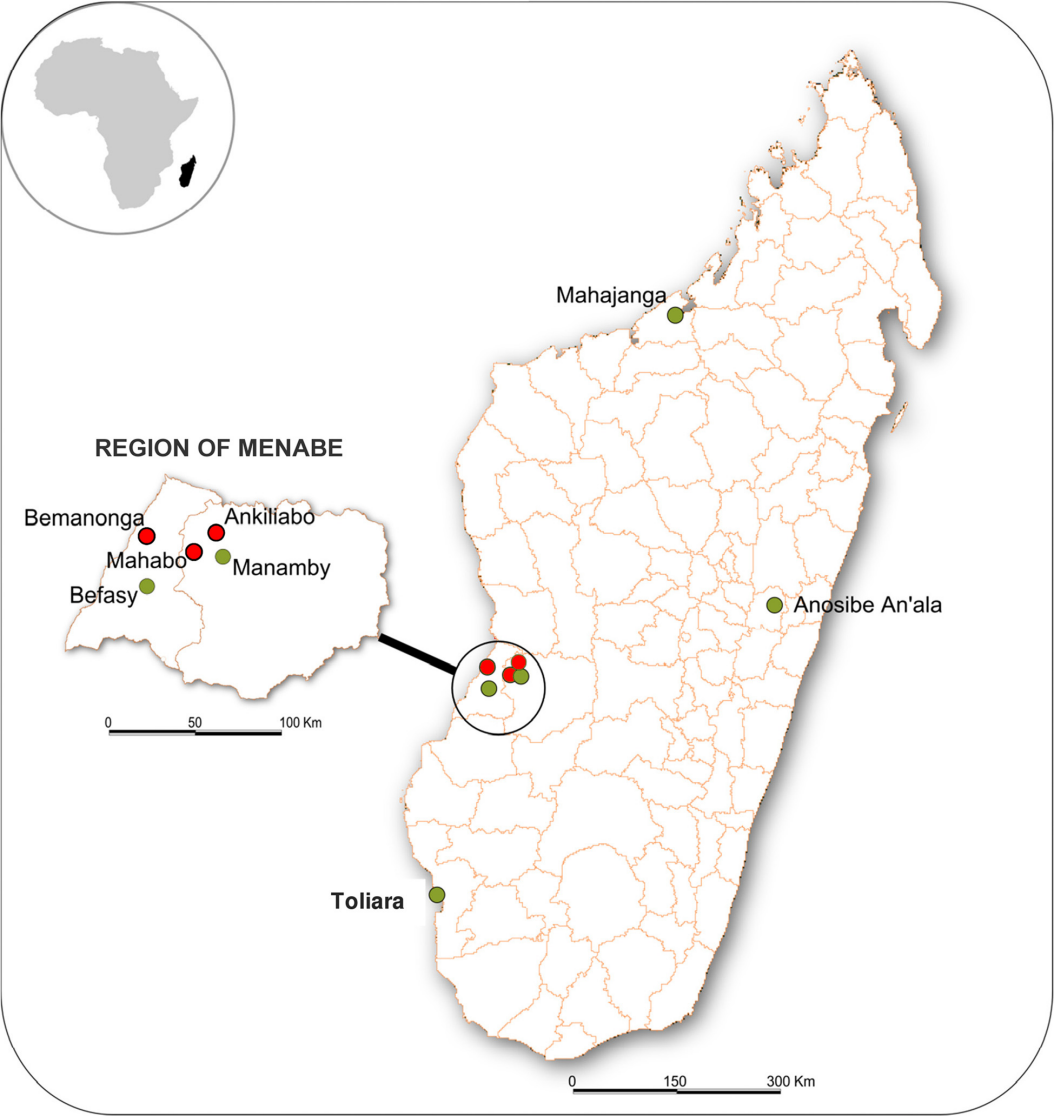
**Location of sampling collection.** Map of Madagascar showing sites of bats capture (green circle) and sites from the Region of Menabe where at least one CoV was detected from locally occurring bats (Red circles).

A total of 351 bats belonging to 3 endemic bat species of the family Pteropodidae were captured and sampled: *Rousettus madagascariensis* (n = 179), *Pteropus rufus* (n = 76) and *Eidolon dupreanum* (n = 96) (Table 1). None of the throat swabs from any bat species (n = 265) tested positive for CoV, but 4.5% (14/313) of fecal specimens tested positive for CoV. Prevalence within *P. rufus*, *E. dupreanum* and *R. madagascariensis* was respectively 17.1% (13/76), 1.0% (1/96) and 0% (0/141). All positive specimens originated from bats captured in the Menabe Region (Figure 1).

**Table 1:** **Geographic location, number of bats tested and number of samples positive for coronavirus in Madagascar**

**Sampling region/site**	**Geographic location**	**Period of capture**	**Nb. bats captured**	**Species**	**No. samples tested throat/fecal**	**No. samples positive throat/fecal**
**Toliara**	Southwest	Feb. 2007	85	*Eidolon dupreanum*	0/85	0/0
**Anosibe An’ala**	Northeast	Feb. 2010	55	*Rousettus madagascariensis*	55/19	0/0
**Menabe**	Southwest	Jun. 2010 Jul. 2011				
Manamby			3	*Pteropus rufus*	3/3	0/0
Befasy			3	*Pteropus rufus*	3/3	0/0
Ankiliabo			48	*Pteropus rufus*	48/48	0/5
			1	*Eidolon dupreanum*	1/1	0/0
Mahabo			9	*Pteropus rufus*	9/9	0/2
			8	*Eidolon dupreanum*	8/8	0/1
Bemanonga			13	*Pteropus rufus*	13/13	0/6
			2	*Eidolon dupreanum*	2/2	0/0
**Anjohibe**	Northwest	Nov. 2010 Nov. 2011	124	*Rousettus madagascariensis*	123/122	0/0
**Total**			351		265/313	0/14

Short amplicon sequences of 329 bp in length of the RdRp gene were obtained for all PCR-positive animals, whereas larger fragment of 993 bp sequences could only be obtained from seven of the 14 PCR-positive animals.

### Phylogenetic analysis

**Figure 2:**
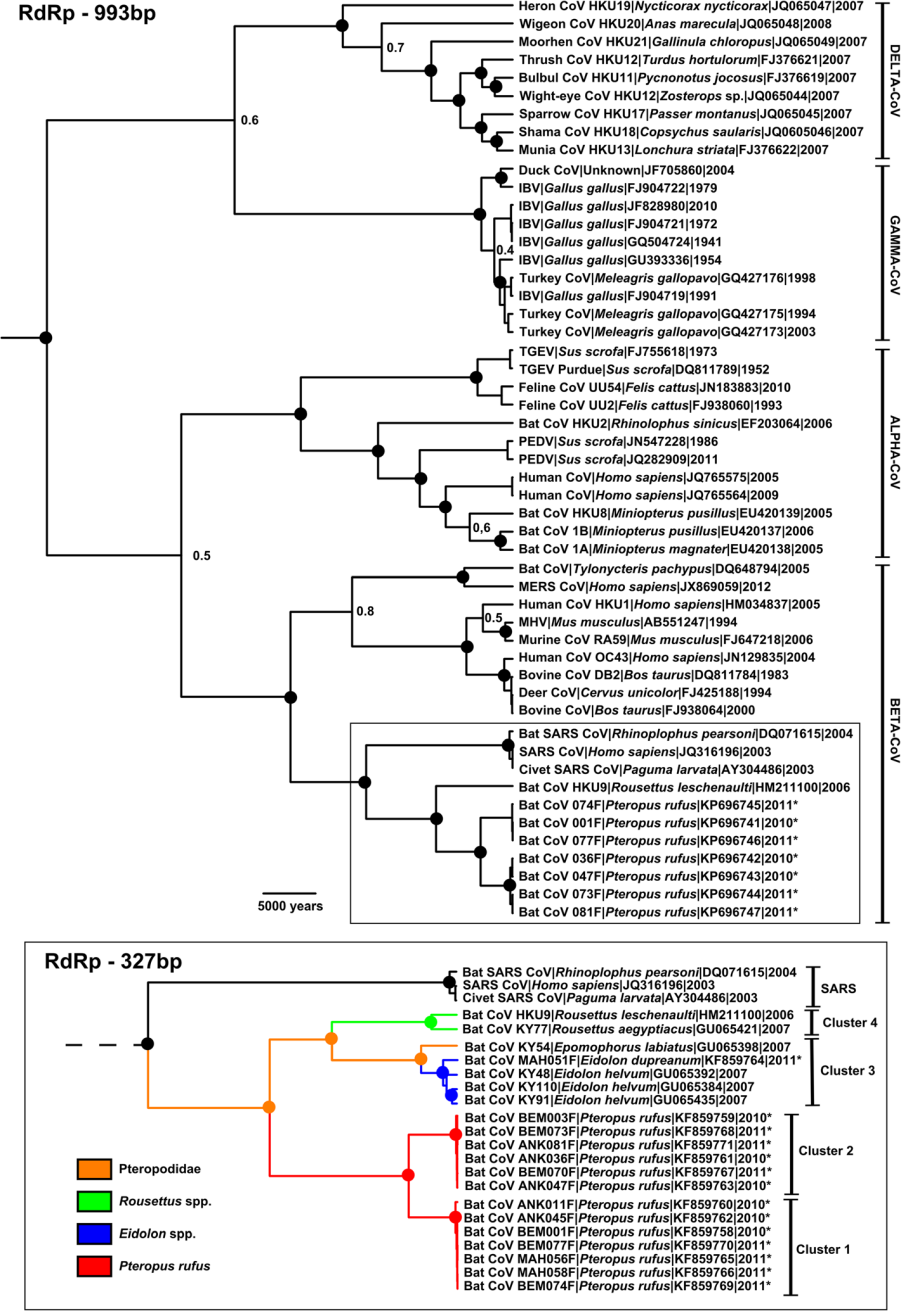
**Bayesian phylogenetic tree generated using 993 nucleotides of the RdRp gene sequences of Malagasy bat CoVs and reference strains of CoVs group.** Major viral genera are shown. Strains detected in this study are marked with an*. Posterior probabilities superior to 0.9 are indicated by a dot, other posterior probabilities are indicated in decimal form to the right of each node, and some have been left off for clarity. The scale bar is expressed in years. The inset figure shows the partial phylogeny of the highlighted region of the *Betacoronavirus* subgroup, including additional reference sequences and with a total alignment length of 329 nucleotides. Techniques used for generating each phylogenetic representation were identical, as detailed in the materials and methods section.

All amplicon sequences were aligned in-frame with a compilation of reference sequences from GenBank for which collection-date data was available [28], giving final alignments containing 51 different sequences of 993 bp in length and 64 different sequences of 329 bp in length. GTR + I + G was identified as the optimal substitution model using jModeltest v2.1.2 [29]. Multiple phylogenies were generated in BEAST using different combinations of model parameters, and the best models were selected using the Tracer [30]. Bayes factor analysis employing marginal likelihoods, as detailed in [12]. All parameter combinations produced identical, strongly supported tree topologies (data not shown). As has elsewhere been determined by Lau et al. [12], Bayesian skyline using a relaxed, exponentially distributed clock model was found to be the best fitting model for RdRp dated-tip phylogenies. The final phylogenetic analyses (Figure 2) revealed that strains from Madagascar are members of the *Betacoronavirus* genus, rooting with Hong Kong strain BtCoV-HKU9 (HM211100) and Kenyan strain KY77 (GU065421), with posterior probabilities of 1. These lineages could be described as SARS-like, and were uniquely affiliated with frugivorous bat hosts of the family Pteripodidae. Malagasy strains were sub-divided into three distinct clusters: two of which were closely related (clusters 1 and 2) and originating from *P. rufus*, and one more distantly related (cluster 3) containing a strain detected from *E. dupreanum* and sequences previously detected from *E. helvum* in Kenya [21].

The Malagasy CoVs were detected from bats captured in three different sites of the Menabe region (West of Madagascar). Within cluster 1, strains were originated from Bemanonga, Mahabo and Ankiliabo. Within cluster 2, strains were originated from Bemanonga and Ankiliabo. The only virus detected belonging to cluster 3 was detected in one bat captured in Mahabo. Overall, identities among Malagasy CoVs ranged from 65 to 100% at the nucleotide level and 70.9 to 100% at the amino acid level (data not shown).

Molecular dating estimates based on the 993 bp fragment of the RdRp gene estimated the timescale of evolution of the coronavirus family to be thousands to tens of thousands of years, however dating estimates proved inaccurate, with broadly spanning HPD values at individual node positions.

## Discussion

In the context of this study, we detected 14 coronaviruses forming nine genetically distinct strains in two endemic Malagasy frugivorous bat species. The overall prevalence (4.5%) is consistent with those identified in studies elsewhere [31-33]. Thirteen viruses were detected from *Pteropus rufus* and 1 virus from *Eidolon dupreanum*. We did not detect CoVs among the sampled *R. madagascariensis*. The detection of novel bat CoVs supports the observation that these viruses are diverse and have a nearly worldwide distribution [17,32,34,35].

We observed that all Malagasy bat CoVs detected in the present study belong to a SARS-related subgroup of the *Betacoronaviruses*, with relatively close homology to BtCoV-HKU9 [36]*.* Our strains displayed 3 distinct clusters: 2 clusters associated with *P. rufus* and 1 cluster associated with *E. dupreanum*. It can be inferred from the results that multiple clusters of CoVs occurring within Malagasy bat populations co-circulate and possibly in a syntopic manner. The high nucleotide and amino acid divergence between clusters and compared to the reference virus BtCoV-HKU9 suggests previously undescribed genetic lineages. Given the mobility of bats, and the especially long distances that can be travelled by colonies of fruit bats [37], these coronaviruses may be spread over a large region. However, host-genus-specific phylogenetic clustering (Figure 2, inset) suggests likely host-specificity which may limit viral spill-over. Thus, further molecular epidemiology studies would be required to fully understand the dispersal potential of CoVs amongst Malagasy bats species.

It is important to remember that, although all three of Madagascar’s fruit bat species were sampled, nothing is known of CoV dynamics in tropical fruit bats, and many factors such as seasonality, bioclimate and the presence of other host species may have important influences on CoV prevalence in these populations. More studies are needed in different locations including different species, particularly those with an insectivorous diet, to reveal a more comprehensive view of the diversity of these viruses in Madagascar. Since the strains of *Betacoronavirus* identified from Madagascar are closely related to those known from Africa, some preliminary biogeographic considerations are in order. All three bat species analyzed herein are endemic to Madagascar. The genus *Pteropus* has a broad distribution from the Australia-New Guinea region west across the Indian Ocean to offshore islands of Tanzania; it is unknown from the African continent. The genus *Eidolon* is composed of two species: *E. helvum* occurring on the African mainland and offshore islands and *E. dupreanum* restricted to Madagascar. Based on a phylogeographical study, both species show broad panmictic population structure [38]. Further, these two taxa are estimated to have diverged from one another sometime in the late Miocene or early Pliocene [39]. The genus *Rousettus* is broadly distributed across Africa and Asia and the ancestral origin of the Malagasy species, *R. madagascariensis*, is unresolved [40]. As with the other two Pteropodidae species occurring on Madagascar, *R. madagascariensis* shows little genetic population structure and presumably broadly disperses across the island, which in turn has important epidemiological implications for these bats transmitting different zoonoses.

Although estimates of the most recent common ancestors (MRCAs) proved inaccurate in our study, most likely as a result of a limited sequence availability from the identified viral strains, standard evolutionary analyses have estimated CoV origins to date to somewhere between 500–10,000 yrs. in the past [41-43]. Nevertheless, further investigations into the relevance of MRCA prediction methodologies are required and a great level of caution must be employed in the interpretation of MRCA data. .

Alternatively, viral lineages may have been imported to Madagascar in recent history: While the vast majority of the island’s bat fauna is endemic, a few species apparently disperse across the Mozambique Channel. Probably the best example is the Molossidae species *Mops midas*, for which, based on genetic data, Southern African animals are nested within Malagasy populations [44]. This bat makes its day roosts in rock crevices and may broadly occur synoptically at such sites with *E. dupreanum* and *R. madagascariensis*. These two fruit bat species are known to feed in the same fruiting trees with *P. rufus* [45], which would complete the cycle of how CoVs originated from Africa mainland could be carried to Madagascar and transmitted to different species of pteropodid bats.

Although we were not able to evaluate risk of human infection, the strains detected here may be considered as potential human pathogens, as bats are natural reservoirs of some pathogenic CoVs. Isolation of Malagasy CoVs using cell culture and molecular analysis of spike (S) gene could better evaluate risk of human infection. Also, a longitudinal study amongst people who frequently handle live bats (e.g. bat hunters, bat bushmeat purveyors, and scientists), and who represent populations at higher risk of infection, would be interesting to establish possible cases of transmission to humans and public health risks.

## Conclusions

In our study, we confirm that CoVs are circulating in two species of endemic bats in Madagascar. Further work on CoV diversity amongst the island’s bat species, as well as aspects of the ecology and behavior of susceptible taxa, are needed to understand the origin, evolution and dispersal of these viruses across the island. To conclude, the results of our study demonstrate the need to develop research programs that aim at surveying viruses in the wild, especially in bats, in order to address possible emergence of zoonotic viruses within human populations.

## Methods

### Study sites

We sampled frugivorous bats in four different areas of Madagascar: Anjohibe, Anosibe An’Ala, Menabe and Toliara (Table 1/Figure 1) based on known accessible colonies of roosting bats and sites where bats are frequently hunted and eaten by people. In the region of Menabe, we selected 5 different sites situated at a mean distance of 28 km around Mahabo to capture and collect specimens, while in the three other regions, sampling occurred at a single site. Sampling was carried out under protocols approved and permitted by Ministry of Environment and Forest (Authorization # 301/08, 101/09, 163/10, 032/11 and 261/11).

### Capturing and sampling

Fruit bats were captured by the use of mist-nets set near roosting sites (trees or caves) and with help of professional hunters [46]. Rectal and throat swabs were collected from each individual bat*.* All bats were identified according species specific morphological features well known by our field trained team (ecologist and veterinarian) and subsequently released. Swabs were placed in viral transport media, almost immediately conserved in liquid nitrogen in the field and stored at −80°C upon arrival at the laboratory in Antananarivo.

### RNA extraction

RNA was extracted from specimens using the QIAamp Viral RNA minikit (QIAGEN, Courtaboeuf, France) according to the manufacturer’s protocol. Briefly, total RNA was extracted from 140 μL of each sample and eluted in 65 μL of Qiagen AVE elution buffer. The extracted RNA was either immediately analyzed or stored at −80°C until use.

### Viral amplification and detection

Extracted RNA was reverse transcribed to generate cDNA by using the M-MLV Reverse Transriptase (Invitrogen, California, USA) into a 2 step reactions. First, 2 μL of RNA were mixed in a solution containing 0.5 μM of random hexamer primers (Roche Diagnostics, Mannheim, Germany), 1 U/μL of RNase inhibitor (10 000 units) and 8.5 μL of water, at 80°C for 5 min, 50°C for 5 min and 4°C for 15 min. Then, 8 μL of RNA issued from the first step was added to a mixture of 0.5 mM of each dNTP (deoxynucleotide triphosphates), 10 U/μL of reverse transcriptase M-MLV, 1 X of buffer and 0.01 M of DTT (dithiothreitol), and incubated at 42°C for 50 min and 95°C for 5 min.

PCR assay was performed to amplify the RNA-dependent RNA polymerase (RdRp) gene which is highly conserved in all known coronaviruses [47]. The primers pair (Forward 5′-GGTTGGGACTATCCTAAGTGTGA-3′; Reverse 5′-CCATCATCAGATAGAATCATCATA-3′) was designed to amplify a 440 bp fragment as described previously [47]. Reaction mixture was carried out using the GoTaq/dNTP mix, Custom kit (Promega Corporation, Madison, USA). Briefly, 5 μL of cDNA was mixed with 1 X of Green GoTaq Flexi Buffer, 1.5 mM of MgCl_2_, 0.2 mM of each dNTP, 0.2 μM of each primer, 0.025 U/μL of GoTaq DNA Polymerase and 28.75 μL of water nuclease-free giving a final volume of 50 μL. The thermocycling was performed under the following conditions: activation at 95°C for 10 min and 40 cycles of denaturation at 95°C for 1 min, annealing at 58°C for 1 min, extension at 72°C for 1 min, and a final extension at 72°C for 5 min. All negative samples were tested in a semi-nested PCR with the same PCR program and using the following pair of primers (Forward 5′-GGTTGGGACTATCCTAAGTGTGA-3′; Reverse 5′- ATCAGATAGAATCATCATAGAGA-3′). Amplicons products were subsequently electrophorezed on a 2.0% agarose gel and visualized using ethidium bromide under UV light. All specimens that showed a positive band at the expected size (440 bp) were sequenced on both strands by Beckman Coulter Genomics (Essex, UK).

From the sequences obtained from the 440 bp, fragment, we designed new primers (reverse) that were strain specific for Malagasy BatCoV (5′-GATGACCTGTATATTCCCA-3′ and 5′-ATGACCTATACATACCCATG-3). We then amplified a large fragment of the RdRp gene by using the consensus forward primer 5′-GTGTACGCTGCTGATCCTGCTATGCA-3′ [48]. The following conditions were performed: 95°C for 10 min and 40 cycles of denaturation at 95°C for 1 min, annealing 56°C for 1 min, extension at 72°C for 1 min, and a final extension at 72°C for 5 min. The final size of sequences used for molecular dating was 1086 bp.

### Sequences and phylogenetic analysis

Sequences from the 440 bp or 1086 bp fragments of the RdRp gene were cleaned and aligned with reference sequences collected from a literature search. Alignment was performed using the translation alignment tool in Geneious pro™ v.6.3.2, created by Biomatters (available from http://www.geneious.com/), and the default ClustalW cost matrix. The final alignment was respectively 329 bp and 993 bp in length for fragments 440 bp and 1086 bp, and contained no free-end or internal gaps. From these alignments, the appropriate substitution model was identified in jModeltest v.2.1.2 [29,49]. Using the appropriate substitution model, 2*10^8 iterations were run with or without the use of a 3-base codon model, using different clock models, alternating between constant and Bayesian Skyline population size models, and seeding with uncorrelated log-normally distributed priors. Trees were sampled every 2x10^5 iterations, and analysis convergence was assessed in tracer v.1.4.0 [50] (available from http://beast.bio.ed.ac.uk/tracer). All analyses converged after a 10% burn-in to give Effective Sample Size values for all parameters superior to 200. Bayes factor analyses were performed in Tracer v1.4.0, with 1000 bootstrap replicates to assess the relative performance of model selections on the generated phylogenies. After identification of an optimal model for phylogenetic classification and dating, two further independent analyses (2*10^8 iterations, sampling every 2*10^5 iterations) were run in BEAST, and all analyses were combined in logCombiner (BEAST package) with a burn-in of 10%, leaving only converged parameter estimates. The final phylogeny and MRCA for fragments 993 bp in length dates were extracted using TreeCombiner (BEAST package) and FigTree v.1.4.0 (available at http://tree.bio.ed.ac.uk/software/figtree/).
